# Determining the shelf life and quality changes of potatoes (*Solanum tuberosum*) during storage using electronic nose and machine learning

**DOI:** 10.1371/journal.pone.0284612

**Published:** 2023-04-28

**Authors:** Ali Khorramifar, Mansour Rasekh, Hamed Karami, Jesús Lozano, Marek Gancarz, Ewa Łazuka, Grzegorz Łagód

**Affiliations:** 1 Department of Biosystems Engineering, University of Mohaghegh Ardabili, Ardabil, Iran; 2 Department of Petroleum Engineering, College of Engineering, Knowledge University, Erbil, Iraq; 3 Escuela de Ingenierías Industriales, Universidad de Extremadura, Badajoz, Spain; 4 Institute of Agrophysics, Polish Academy of Sciences, Lublin, Poland; 5 Faculty of Production and Power Engineering, University of Agriculture in Kraków, Kraków, Poland; 6 Faculty of Technology Fundamentals, Lublin University of Technology, Lublin, Poland; 7 Faculty of Environmental Engineering, Lublin University of Technology, Lublin, Poland; Sultan Qaboos University, OMAN

## Abstract

The activities of alpha-amylase, beta-amylase, sucrose synthase, and invertase enzymes are under the influence of storage conditions and can affect the structure of starch, as well as the sugar content of potatoes, hence altering their quality. Storage in a warehouse is one of the most common and effective methods of storage to maintain the quality of potatoes after their harvest, while preserving their freshness and sweetness. Smart monitoring and evaluation of the quality of potatoes during the storage period could be an effective approach to improve their freshness. This study is aimed at assessing the changes in the potato quality by an electronic nose (e-nose) in terms of the sugar and carbohydrate contents. Three potato cultivars (Agria, Santé, and Sprite) were analyzed and their quality variations were separately assessed. Quality parameters (i.e. sugar and carbohydrate contents) were evaluated in six 15-day periods. The e-nose data were analyzed by means of chemometric methods, including principal component analysis (PCA), linear data analysis (LDA), support vector machine (SVM), and artificial neural network (ANN). Quadratic discriminant analysis (QDA) and multivariate discrimination analysis (MDA) offer the highest accuracy and sensitivity in the classification of data. The accuracy of all methods was higher than 90%. These results could be applied to present a new approach for the assessment of the quality of stored potatoes.

## Introduction

Potato (*Solanum tuberosum*), as a resistant plant, grows in most parts of the world. It is compatible with dryness, harsh temperature, and soils with lower fertility [1]. Potatoes (*Solanum tuberosum*) contain high levels of starch, various vitamins, proteins, and mineral salts, such as calcium, phosphorous, and iron at low calories. Owing to such properties as improvement of the immune system and cancer prevention, this crop is widely used, either fried or cooked [2, 3]. Its consumption is increasing due to the abundance of useful nutrients such as carbohydrates, dietary fiber, minerals, and other health-promoting compounds, such as beta-carotene, vitamin C, and phenolic acids [4]. This crop is also rich in polyphenols, chlorogenic acids, isochlorogenic, caffeic, cinnamic, and hydroxycinnamic acids. It can act as a functional component of health-enhancing foods to prevent cancer, diabetes, and inflammation. Long-term consumption of foods based on sweet potatoes can indeed contribute to the improvement of human health [5].

Conventional methods for evaluating the quality of potatoes are mostly destructive and inefficient. In the practical production of potatoes, the quality evaluation system must be characterized by good accuracy, high speed, and low cost. Such goals can be achieved through modern techniques, such as spectroscopy, thermography, acoustics, and electronic nose, because they do not require sample preparation, while being non-destructive, efficient, fast, accurate, pollution-free, and inexpensive [6, 7]. In the meantime, due to its different structure and approach from other non-destructive methods, electronic nose can be highly effective in determining the quality of cultivars. Because in other non-destructive methods, more than one factor may affect the output information (which will lead to an increase in error) and they will need strong calibration, which electronic nose is an exception to this rule [8]. Also, conventional methods are very boring, hard and expensive (due to lack of automatic control) and are influenced by environmental factors [9–11]. But the electronic nose covers all these disadvantages.

Food quality and safety are the major concerns for human health [12]. The demand for green, bulk, and healthy food is sharply increasing. Food quality is affected by several factors, such as light, temperature, gases, aromas, relative humidity, and the presence of microorganisms[13, 14]. The proliferation of microorganisms is the main cause of spoilage and deterioration of materials [15].

During the storage of sweet potatoes, the activities of alpha-amylase, beta-amylase, sucrose synthase, and invertase are affected by storage conditions, which can impact the structure of starch and reduce the sugar content, hence altering the quality of sweet potatoes [16]. Polyphenols also interact with enzymes and starches, as well as modulate the overall digestion of starch [16, 17]. The tuber is a major harvested organ of the plant and is mainly involved in storing and reproducing nutrients. The cultivation of sweet potatoes is seasonal. Potatoes are usually planted in spring and early summer, while they are harvested in autumn and early winter. After harvest, tubers are stored for several months. Therefore, optimal storage conditions play a key role in post-harvest quality management. In general, the temperature range of 3.8 to 10°C and the relative humidity (RH) of 90 to 95% are optimal conditions for long-term storage of potatoes [18]. However, tubers are large, characterized by high humidity, high respiratory activity, thin skin, as well as delicate flesh and delicate texture, which makes them vulnerable during storage. The post-harvest storage of this crop has negative consequences for fresh and processed sweet potato crops, such as moisture loss, changes in the nutrient composition, or soft rot spoilage caused by pests and diseases that can seriously affect quality and reproduction [19]. Long-term storage of potato tuber crop is associated with quantitative changes involving, inter alia, reduction of the original weight of tubers due to transpiration, respiration, and sprouting. Transpiration, respiration, and sprouting processes are mainly determined by the relative humidity of air and temperature during storage. Meteorological conditions during the vegetation season, genetic characteristics of varieties, and mechanical damage to tubers caused during harvesting and post-harvest treatment may also be responsible for natural losses of the potato tubers [20].

Volatile organic compounds (VOCs) are a key indicator in assessing fruit quality and are also an important consideration for customers [21]. Analysis of VOCs is a possible way to determine the quality of fruit. The electronic nose (e-nose) is a widely used olfactory system, extensively employed for the detection of VOCs [22]. Owing to its rapid and simple operation, as well as non-destructive and cost-effective detection, the e-nose has attracted a lot of attention. Thus far, e-nose systems have been applied for classifying fruit, predicting the quality of peaches [23], apples [24], apricots [25], mandarins [26], berries [27], sweet cherries [28], mint [29], rice [30] and mangoes [31]. However, the e-nose technology has been rarely used to investigate the qualitative changes of agricultural products during the storage period. Moreover, most of the previous works on fruit quality testing by the e-nose technology were based on destructive sampling, in which the fruit was cut to obtain more VOCs [32–34]. However, in the real fruit supply chain, VOCs must be detected from intact fruit with no destructive sampling.

The purpose of this article is to detect the shelf life of several potato (*Solanum tuberosum*) cultivars as well as their SSC and carbohydrate changes during storage using electronic nose and PCA, LDA, SVM, and ANN methods in a non-destructive way (without the need to conduct chemical tests) and Faster than conventional methods.

## Materials and methods

### Sample preparation

This study was carried out on three potato (*Solanum tuberosum*) cultivars (Sprit, Santé, and Agria) that were harvested from the fields of Ardabil Agricultural Research Center. Potato harvest time was in November 2021. At this time, the average temperature was 5–15°C, and the air humidity was 80–90%. After that, the potatoes were transferred to the cold store, where the temperature and humidity were 6°C and 90–95%, respectively. To standardize the conditions of heat and mass transfer with the environment during storage and to minimize the effect of possible differences in temperature and humidity, 10 kg of each variety were stored in wooden boxes in single layer, and the free spaces between the boxes were of similar size. After storing the potato samples in the warehouse, the data were collected 5 times at 2-week intervals. In total, the research data included 5 storage periods and 3 potato cultivars with 18 replications.

### SSC measurements

In each period of storage, the potato tubers were first washed and peeled, as the soluble solid content (SSC) of the potato flesh was considered (not the flesh part with the skin). After peeling, the sample was grated to squeeze its water into the microtubes. The SSC of potatoes was measured by a BPTR100 liquid refractometer model (Middle East System Control Company under the brand name of Prisma Tech, made in Iran) (obtained according to the BRICS index). These experiments were performed in 18 replications for each potato cultivar and storage period.

### Measurement of carbohydrates

To measure the carbohydrate content of the potato during the storage period, the sample was prepared by means of the Schlegel method. Typically, 0.2 g of potato tubers was heated to 80°C with 10 cc of ethanol 95% in a Benmary bath for one hour. Then, 1 cc of phenol 0.5% and 5 cc of 98% sulfuric acid were added to each cc of this sample. The prepared sample was then placed in a nano-spectrophotometer (Nanodrop) with a volume of 1000 microliters using a cuvette (manufactured by the Termo scientific company, USA) to determine its optical absorbance. Afterwards, the carbohydrate content was determined (μg/ml) based on optical absorption and standard curve [5].

### Electronic nose

Data collection was conducted by an olfactory machine (in the Department of Mechanical Engineering of Biosystems of Mohaghegh Ardabili University of Iran) [35, 36]. The above-mentioned electronic nose encompassed 9 metal oxide sensors (MOS) the specifications of which are listed in Table 1.

**Table 1:** Specifications of sensors used in the e-nose [37, 38].

Sensor No.	Sensor	Target gas	Sensitivity (PPM)
1	MQ9	CO and combustible gas	10–1000 and 100–10,000
2	MQ4	Urban gases and methane	300–100
3	MQ135	Steam ammonia, benzene, sulfide	10–10,000
4	MQ8	Hydrogen	100–1000
5	TGS2620	Alcohol, steam organic solvents	50–5000
6	MQ136	Sulfur dioxide (SO_2_)	1–200
7	TGS813	CH4, C3H8, C4H10	500–10,000
8	TGS822	Steam organic solvents	50–5000
9	MQ3	Alcohol	10–300

**Fig 1:**
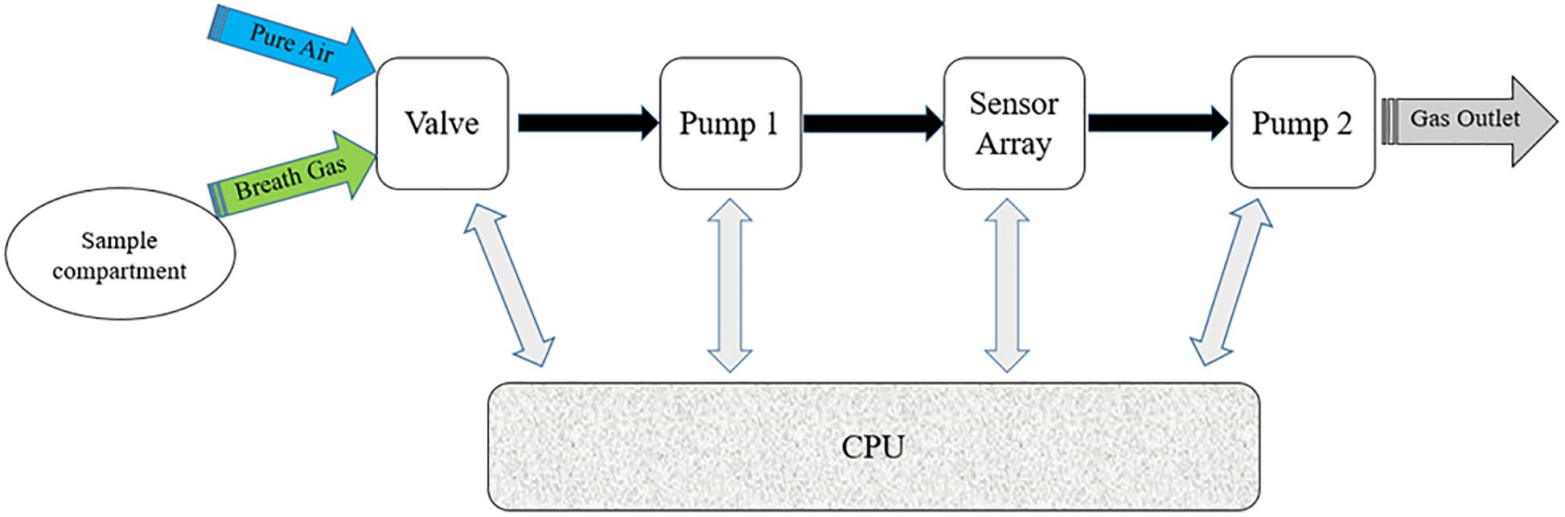
Diagram of the hardware part of the e-nose.

The hardware part of the e-nose device includes the valve, pump, sample chamber, air valve, sensor array, the main processor board, and the control circuit, the diagram of which can be found in Fig 1.

The condition for starting the data collection was that the potato samples should be first placed in a closed sample container for 24 hours, so that their aroma and volatile compounds saturate the sample container space. 2–4 potatoes from each cultivar were placed in the sample container with a volume of 1000 ml. The sample chamber was then connected to the e-nose and the data collection operation began. In the first step, fresh air was passed through the sensor chamber to clean the sensors from the odors and other compounds, then the odor of the potato inside the sample chamber was pumped to the sensors chamber; finally, clean air was injected into the sensor chamber to prepare the system for subsequent data collections (each of these steps took 100 s). All these operations were online and automatic. The data of each sample were collected in 15 replications, considering 3 potato cultivars and 6 time periods, each sensor contained 270 raw data sets. The experiments were carried out in 6 periods at 15-day intervals.

### Machine learning methods

#### PCA method

The principal component analysis method (PCA) is a method for the reduction of data dimensionality [39, 40]. It can not only increase the interpretability of data by creating non-correlated variables, but it also minimizes data loss [41]. Here, each principal component is a linear combination of all the primary variables. This method also helps to understand the difference of one sample from others (score chart) and which variable has the highest contribution to this distinction [42]. PCA has been used to show the initial relative position of samples in a two-dimensional space to observe the changes in volatiles of potato samples by an e-nose.

#### DA analysis

Discriminant analysis (DA) encompasses the methods that can be used for both classification and dimensionality reduction. Linear discriminant analysis (LDA) is particularly popular, because it is both a classifier and a dimensionality reduction technique [43, 44]. LDA is a generalization of Fisher’s linear discriminant, a method used in statistics for pattern recognition and machine learning to find a linear combination of features that differentiate two or more classes of objects or events [45]. LDA is closely related to PCA, as both seek linear combinations of variables to best describe the data, although LDA usually has a better classification effect than PCA and Cluster Analysis (CA) [46]. Quadratic discriminant analysis (QDA) is a variant of LDA that allows for non-linear separation of data. LDA and QDA are simply two classic classifiers, with a linear and a quadratic decision surface, respectively. Linear discriminant analysis (LDA) uses linear combinations of predictors to predict the class of a given observation. LDA assumes that the predictor variables are normally distributed and the classes have identical variances (for univariate analysis) or identical covariance matrices (for multivariate analysis). QDA is a more flexible method than LDA, because there is no assumption that the covariance matrix of classes is the same. In multivariate discriminant analysis (MDA), each class is assumed to be a Gaussian mixture of subclasses [47].

For e-nose, dicriminant analysis was used to distinguish between different samples, i.a. to determine whether the aroma changes during the storage period were practically detectable by the e-nose.

#### SVM architecture

The support-vector machine (SVM) method aims to find an optimal hyperplane in the multidimensional space of the data, which separates the data points belonging to different classes. Additionally, SVM aims to find a hyperplane with the largest possible margin (distance to the nearest data point) in order to ensure its robustness to new, previously unseen data points. When a new point is observed, SVM predicts its class based on the side of the hyperplane on which the point lays. This method was first introduced by Boser, Guyon and Vapnik [48] and further developed by Cortes and Vapnik [49]; it still remains an active area of research [50, 51]. Several studies have reported that SVM can offer higher classification accuracy than other classification algorithms. In the last decade, SVM has been employed as an important learning technique to solve the classification and regression problems in various fields [45, 52].

#### ANN architecture

The artificial neural network (ANN) algorithm [53] is a machine learning technique that mimics human neural networks and can be used for prediction, classification, clustering as well as pattern recognition based on past and present educational data. ANN introduces multiple neurons organized into layers. A typical network consists of an input layer, one or several hidden layers, and an output layer. Each neuron takes a linear combination of multiple values as input and performs some non-linear transformation of that input, passing the output value forward, as input to the further layers of the network. During the training of an ANN, the weights of the linear combinations are tuned so that the output of the entire network is possibly close to the expected result for all training data points. At the same time, the network should produce good results for previously unseen data points, so the data are usually divided into training, validation, and testing sets [32, 42, 54] in order to evaluate the generalization capability of the network. In this study, the data set for each digit involved 5 time periods; thus, 5 neurons in the output layer, 9 neurons in the input layer, and a hidden layer with 8 neural nodes were selected. During the study, 70% of the data were used for learning, 15% were applied for testing and 15% were used for validation.

The Unscrambler X 10.4 (for PCA, SVM, and LDA) and MATLAB R2016a (for ANN) software was used to perform the calculations.

## Results

### Variation in the quality indices of potato during the storage period

The SSC level represents the sugar content of the fruit. It shows an increase upon ripening by hydrolysis of stored carbohydrates, i.e. starch, to simple sugars by catabolic processes, such as respiration [55]. This metabolic process is often controlled by the activity of phosphorylase or amylase enzymes and has two important consequences: sweetening and softening of fruit tissue. Although the post-harvest moisture loss may also increase SSC due to changes in sugar concentrations, starch decomposition often has a greater effect on its variation [56].

According to the analysis of variance (Table 2), the interaction effect of cultivar × storage period is significant on the SSC changes at the level of 1% and a coefficient of change of 0.15%. For carbohydrates, the interaction of cultivar×storage period is significant on carbohydrate at the level of 5% and with a coefficient of variation of 3.75%.

**Table 2:** Analysis of variance for the chemical parameters of potato.

	Sourse	Degrees of Freedom	Mean Square
SSC	Cultivar	2	7.321
	Storage	4	2.800
	Cultivar × Storage	8	0.093*
	Error	30	0.000
	Total	44	
Carbohydrate	Cultivar	2	41368.945
	Storage	4	9081.722
	Cultivar × Storage	8	140.188**
	Error	30	53.878
	Total	44	

*Significant at p ≤.01

**Significant at p ≤.05

**Fig 2:**
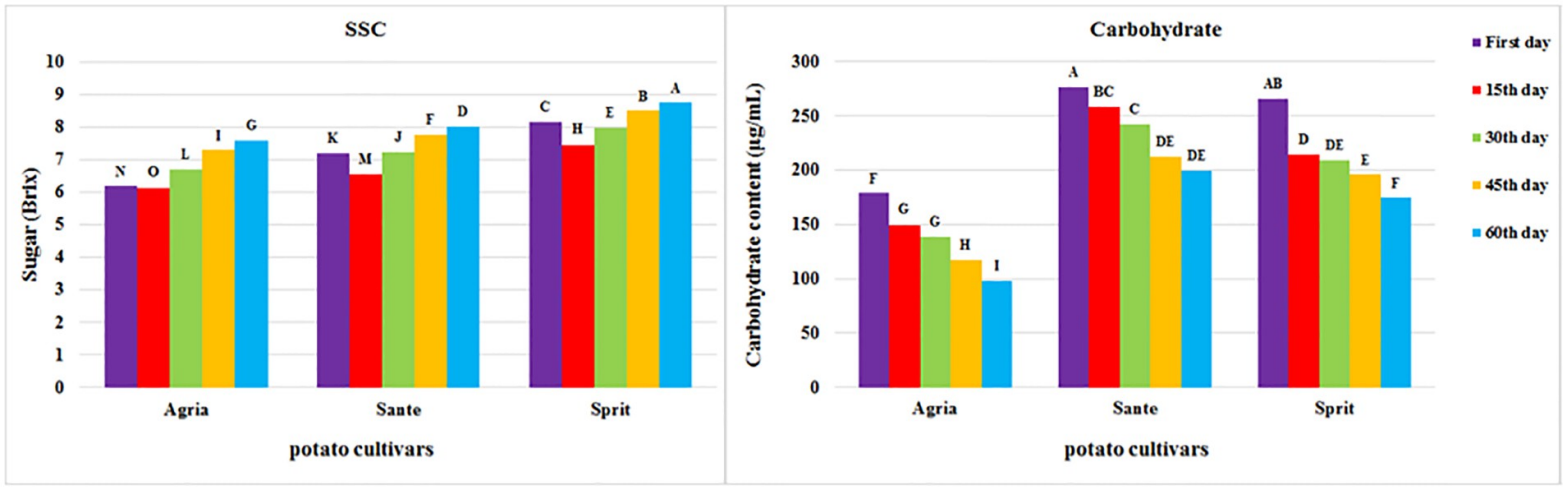
Result of Duncan mean comparison test. Abbreviations: letter A is the highest value and letter O is the lowest value.

Fig 2 presents a comparison of the mean changes of SSC and carbohydrates during storage for three different potato cultivars. According to Fig 2a, the SSC values of the three cultivars were different in all periods, among which the Sprite cultivar always showed the highest value. The Agria cultivar had less SSC than the other two cultivars. The SSC of all cultivars slightly decreased at the beginning of the storage period, followed by an increase over time. Moreover, Fig 2b revealed the varying carbohydrates content of different potato cultivars in all periods. The Santé cultivar exhibited more carbohydrates, while the Agria cultivar had the lowest carbohydrate content. This cultivar also showed lower SSC, among others. The carbohydrate contents of all cultivars decreased during the storage period. In a study by Maruf, Tuhin [57] on the changes in potato nutrients during storage, the decline of sugar was reported.

### PCA results

**Fig 3:**
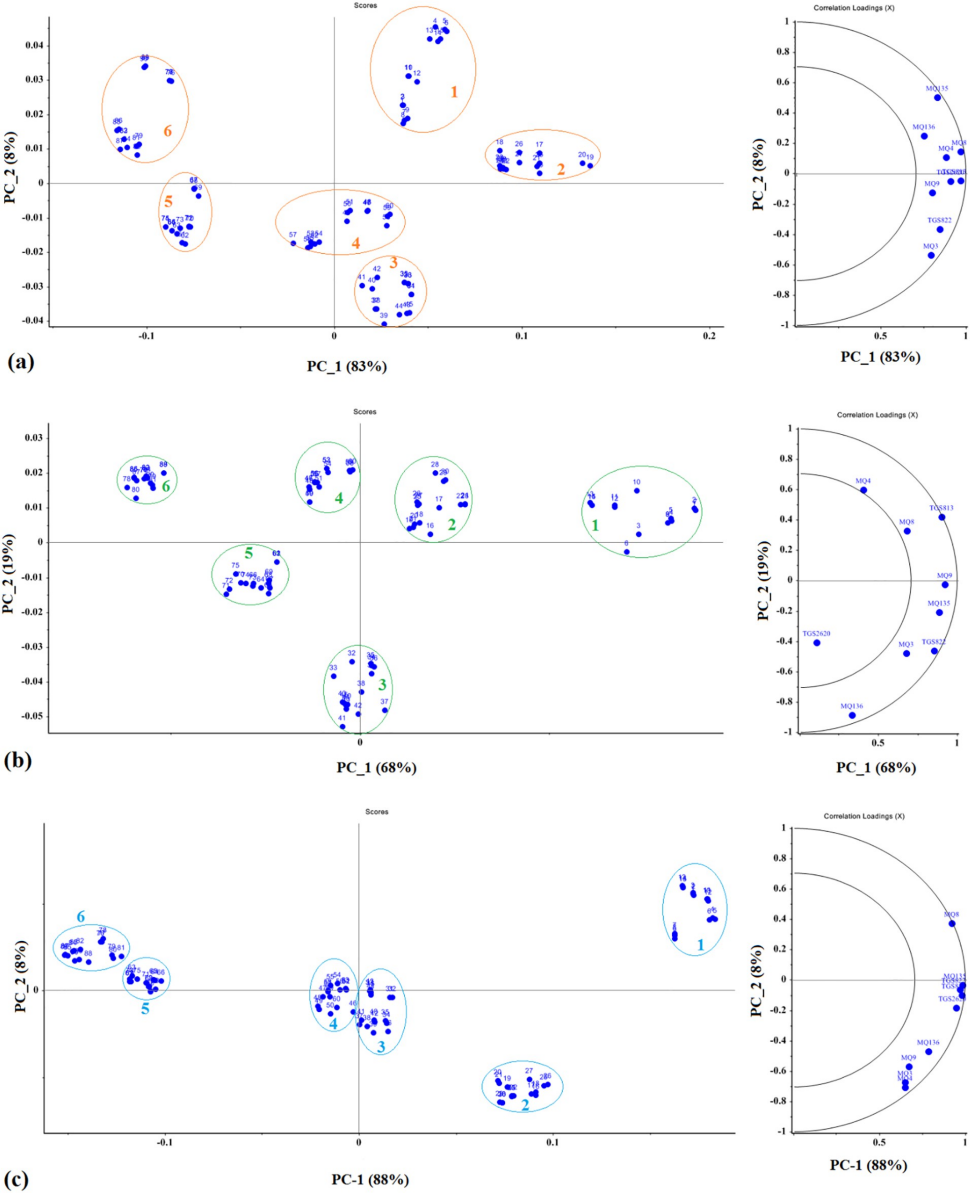
Results of the PCA method for potato cultivars a) Agria, b) Santé, and c) Sprite.

Fig 3 presents the results obtained by means of the PCA method for different potato cultivars. The PCA diagram shows the total variance of the data for the Agria cultivar as PC-1 (83%) and PC-2 (8%), PC-1 (68%) and PC-2 (19%) for the Santé cultivar and PC-1 (88%) and PC-2 (8%) for the Sprite cultivars. Therefore, the first two main components for the Agria, Santé, and Sprite cultivars formed 91%, 87%, and 96% of the total variance of normalized data, respectively. According to Fig 3, all three cultivars underwent a certain process, such that the first data collection period can be observed on the right side of the graph, while the last data collection period (third month) is presented on the left side of the graph. This implies that the data were gradually shifted from right to left of the graph with increasing storage time. Thus, the e-nose responded well to the odor of the cultivars during the storage. On the basis of Fig 3a, all 9 sensors exhibited a good response to the odor of the samples, such that all the sensors were close to the larger circle. The encircle shows 50% and the encircle shows 100% of the variance of the data [38]. For the Santé cultivar (Fig 3b), however, the MQ4T MQ8 and TGS2620 sensors showed a poor response. Fig 3c indicated that the sensors showed a better response than the previous two samples, resulting in an initial classification accuracy of 94%.

**Fig 4:**
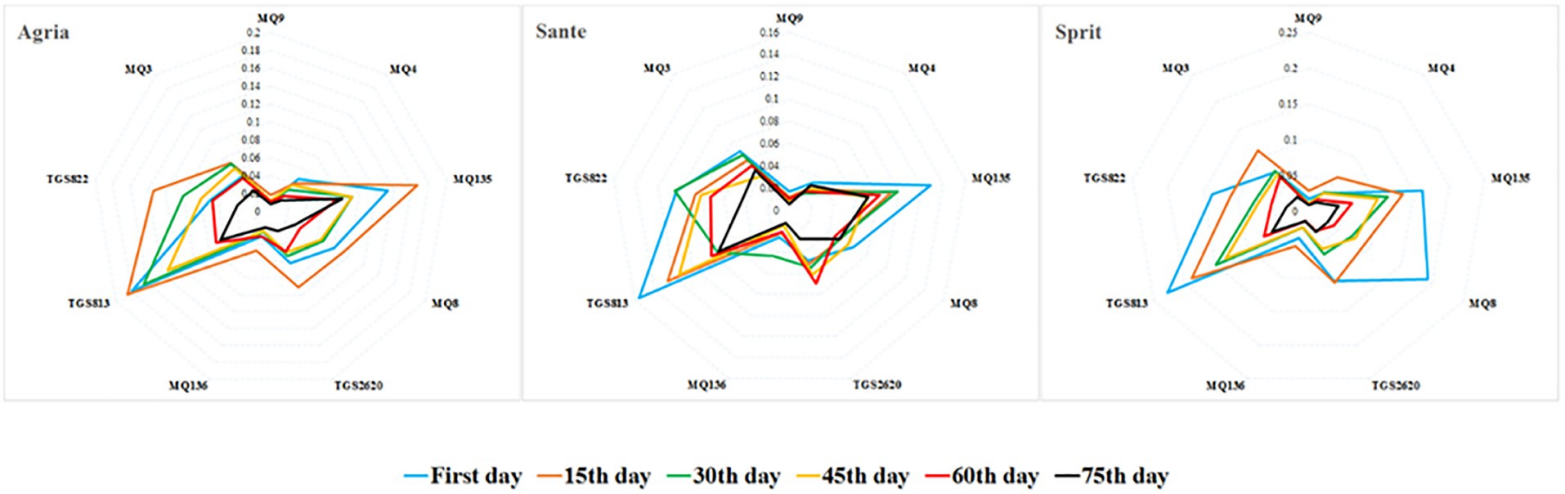
Gas fingerprints of sensor relative-intensity responses to different types of VOCs potatos obtained from sensor array output of e-nose.

As shown in the radar chart (Fig 4), the response of the sensors to the odor of potatoes decreased in all cultivars by the passing of time. The highest response was related to the beginning of the storage period, while the lowest response was observed in the sixth storage period. In general, the highest responses were related to the TGS813 and MQ135 sensors, whereas the MQ136 and MQ9 sensors exhibited the poorest responses.

### DA results

**Fig 5:**
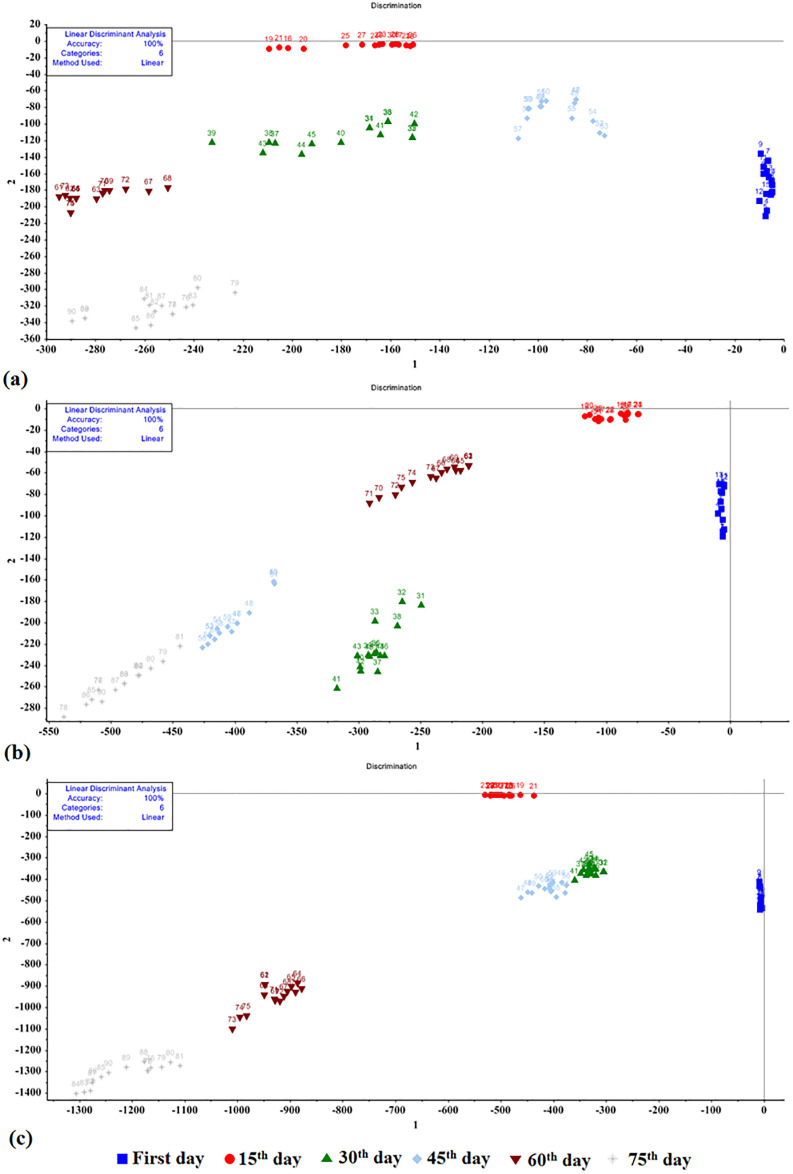
Results of the LDA method for various potato storage periods: a) Agria, b) Santé, and c) Sprite.

The LDA, QDA, and MDA models were utilized to determine the shelf life of potato cultivars using an e-nose (Fig 5). The model inputs were obtained from 9 metal oxide sensors, the weight of the model inputs was one. These methods showed 100% accuracy for all three potato cultivars. The purpose of this method was to determine the quality alterations of each cultivar over time. As shown in Fig 5, all the potato cultivars can be easily differentiated during the storage period. According to the chemical results, the samples showed a similar trend in terms of the carbohydrate content, that is, the samples move to the left side of the graph over time. The left side of the graph indicates a decrease in the aroma of the samples, which may be attributed to the carbohydrate content. Chemical experiments revealed a decline in the carbohydrate contents of all cultivars.

**Fig 6:**
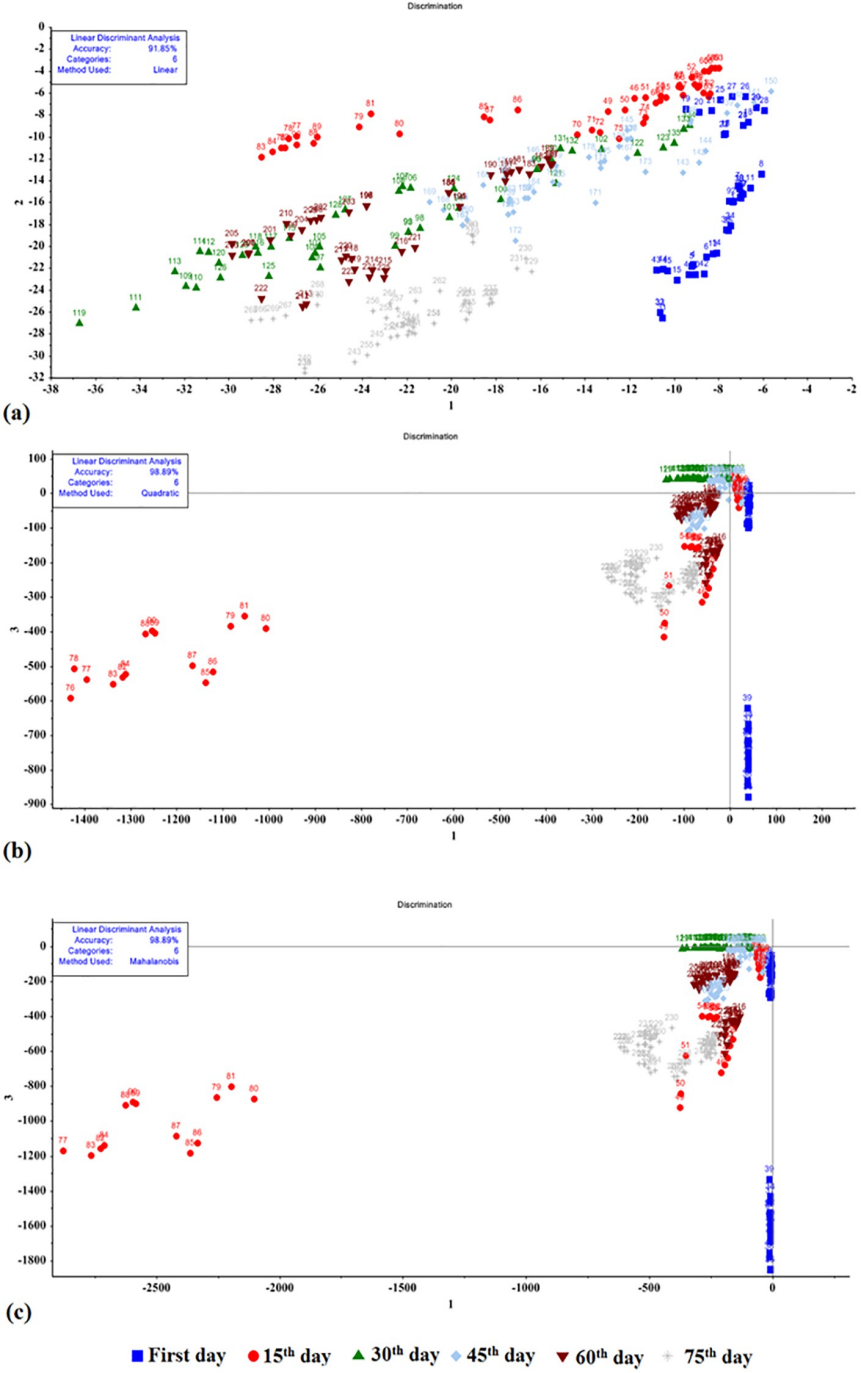
Results obtained using all potato cultivars for three methods: a) LDA, b) QDA, and c) MDA.

An analysis was performed using the LDA, QDA, and MDA models to assess the storage time of potatoes, regardless of their cultivar (Fig 6). This experiment made an attempt not to limit the quality changes to a specific cultivar, as under real conditions, different potato cultivars are stored in the warehouses, emitting various VOCs. Using all data, i.e. 270 data sets obtained by e-nose, these qualitative changes were examined in 6 periods. The results showed that the LDA, QDA, and MDA methods have a total detection of 91.85%, 98.89%, and 98.89%, respectively. The confusion matrix of the performance parameters of the LDA, QDA, and MDA methods is presented in Table 3. The classification performance parameters of the LDA, QDA, and MDA methods can be summarized in Table 4. According to Tables 3 and 4, the QDA and MDA methods led to quite similar results, such that the confusion matrix misclassified 3 out of 270 potato data sets obtained by means of QDA and MDA. The LDA method, however, misclassified 22 data sets. On the basis of Tables 3 and 4, QDA and MDA offered far higher accuracy in data classification compared to the LDA method.

**Table 3:** Confusion matrix for classification of all groups of potato culivar storage using the LDA, QDA, and MDA statistical methods.

Model	storage time	First day	15^th^ day	30^th^ day	45^th^ day	60^th^ day	75^th^ day
LDA	First day	39	0	0	0	0	0
	15^th^ day	6	42	0	0	0	0
	30^th^ day	0	3	40	7	0	0
	45^th^ day	0	0	5	38	0	0
	60^th^ day	0	0	0	0	44	0
	75^th^ day	0	0	0	0	1	45
QDA	First day	45	0	0	0	0	0
	15^th^ day	0	45	0	0	0	0
	30^th^ day	0	0	45	3	0	0
	45^th^ day	0	0	0	42	0	0
	60^th^ day	0	0	0	0	45	0
	75^th^ day	0	0	0	0	0	45
MDA	First day	45	0	0	0	0	0
	15^th^ day	0	45	0	0	0	0
	30^th^ day	0	0	45	3	0	0
	45^th^ day	0	0	0	42	0	0
	60^th^ day	0	0	0	0	45	0
	75^th^ day	0	0	0	0	0	45

**Table 4:** Performance parameters of the LDA, QDA, and MDA models for potato culivar storage classification.

Model	Storage time	Accuracy	Precision	Recall	Specificity	AUC	F score
LDA	First day	0.978	1.000	0.867	1.000	1.000	0.929
	15^th^ day	0.967	0.875	0.933	0.973	0.924	0.903
	30^th^ day	0.944	0.800	0.889	0.956	0.878	0.842
	45^th^ day	0.956	0.884	0.844	0.978	0.931	0.864
	60^th^ day	0.996	1.000	0.978	1.000	1.000	0.989
	75^th^ day	0.996	0.978	1.000	0.996	0.987	0.989
Average per class		0.973	0.923	0.919	0.984	0.953	0.919
QDA	First day	1.000	1.000	1.000	1.000	1.000	1.000
	15^th^ day	1.000	1.000	1.000	1.000	1.000	1.000
	30^th^ day	0.989	0.938	1.000	0.987	0.962	0.968
	45^th^ day	0.989	1.000	0.933	1.000	1.000	0.966
	60^th^ day	1.000	1.000	1.000	1.000	1.000	1.000
	75^th^ day	1.000	1.000	1.000	1.000	1.000	1.000
Average per class		0.996	0.990	0.989	0.998	0.994	0.989
MDA	First day	1.000	1.000	1.000	1.000	1.000	1.000
	15^th^ day	1.000	1.000	1.000	1.000	1.000	1.000
	30^th^ day	0.989	0.938	1.000	0.987	0.962	0.968
	45^th^ day	0.989	1.000	0.933	1.000	1.000	0.966
	60^th^ day	1.000	1.000	1.000	1.000	1.000	1.000
	75^th^ day	1.000	1.000	1.000	1.000	1.000	1.000
Average per class		0.996	0.990	0.989	0.998	0.994	0.989

### SVM results

SVM is one of the most important data mining models which relies on the theory of statistical learning and mathematical optimization, using the principle of minimizing structural error, leading to an overall optimal solution. The C-SVM and Nu-SVM methods were employed to classify the samples. The Nu, C, and γ parameters were validated by trial and error through minimization. In the study, 70% of the data were used for training, while 30% of them were utilized for testing. The input weights were equal to one for all data. Four functions, i.e. linear, sigmoid, radial, and polynomial were applied. The cultivars were first analyzed separately. Then, all cultivars were examined together, regardless of their cultivar type. The results of the SVM method are summarized in Table 5.

**Table 5:** Results and comparison of the Nu-SVM and C-SVM models^1^ subjected to the kernel functions.

Kernel function	C-SVM				Nu-SVM			
	C	γ	Train	Validation	Nu	γ	Train	Validation
Agria								
linear	100	1	100	100	0.225	1	100	100
Polynomial	10	10	85.55	73.33	0.01	1	93.33	92.22
Radial basis function	1	10	90	73.33	0.01	0.01	100	93.33
sigmoid	1	10	62.22	42.22	0.255	0.01	96.67	91.11
Santé								
linear	100	1	100	100	0.01	1	100	100
Polynomial	10	10	74.44	67.67	0.01	0.1	100	97.77
Radial basis function	100	0.1	86.66	73.33	0.745	0.01	100	98.88
sigmoid	10	1	50	41.11	100	0.01	96.66	87.77
Sprit								
linear	100	1	96.66	92.22	0.5	1	100	96.66
Polynomial	100	100	90	82.22	0.255	0.01	100	95.55
Radial basis function	100	1	98.88	88.88	0.745	1	98.88	93.33
sigmoid	100	10	92.22	84.44	0.05	0.01	96.68	86.66
All potato cultivars								
linear	100	1	91.11	88.15	0.225	1	90.37	88.5
Polynomial	10	10	87.77	87.03	0.5	10	90.74	89.62
Radial basis function	10	1	83.70	82.96	0.255	1	87.40	82.22
sigmoid	100	1	90.37	89.25	0.745	0.1	84.44	83.33

^1^ Statistical analysis models and parameters used for data analysis: Nu‐SVM = Nu Support Vector Machine classification, and C‐SVM = C Support Vector Machine classification. Coefficient parameter symbols: c = C‐SVM penalty coefficient; Nu = Nu‐SVM penalty coefficient; γ = core coefficient.

**Fig 7:**
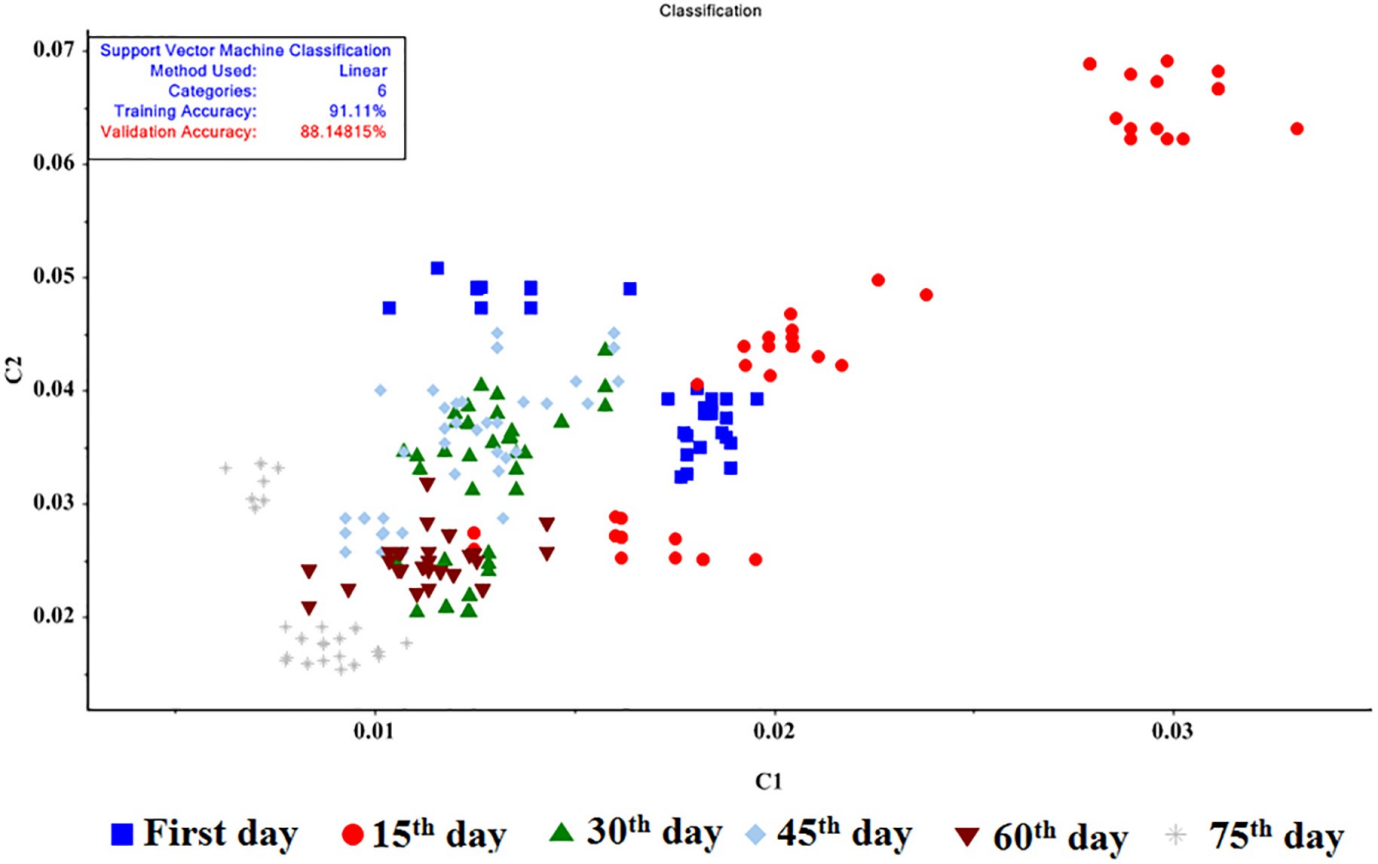
Classification of potato storage period using the C‐SVM (linear).

According to the results obtained for the three potato cultivars, the linear model showed 100% accuracy for training and validation (Fig 7). Using all linear, sigmoid, radial, and polynomial functions, the Nu-SVM method generally offered higher accuracy compared to the C-SVM method. Concerning the classification based on all potato cultivars, the C-SVM method and the linear function exhibited an accuracy of 91.11 and 88.15 for training and validation, respectively. The confusion matrix and performance functions of this model are depicted in Tables 6 and 7.

**Table 6:** Confusion matrix for all group of potato based on storage time classification using the SVM statistical methods.

Model	Storage time	First day	15^th^ day	30^th^ day	45^th^ day	60^th^ day	75^th^ day
C-SVM (linear)	First day	45	0	0	0	0	0
	15^th^ day	0	30	0	0	0	0
	30^th^ day	0	0	40	4	0	0
	45^th^ day	0	15	5	41	0	0
	60^th^ day	0	0	0	0	45	0
	75^th^ day	0	0	0	0	0	45

**Table 7:** Performance parameters of the SVM models for all potato cultivars.

Model	Storage time	Accuracy	Precision	Recall	Specificity	AUC	F score
C-SVM (linear)	First day	1.000	1.000	1.000	1.000	1.000	1.000
	15^th^ day	0.944	1.000	0.667	1.000	1.000	0.800
	30^th^ day	0.967	0.909	0.889	0.982	0.946	0.899
	45^th^ day	0.911	0.672	0.911	0.911	0.792	0.774
	60^th^ day	1.000	1.000	1.000	1.000	1.000	1.000
	75^th^ day	1.000	1.000	1.000	1.000	1.000	1.000
Average per class		0.970	0.930	0.911	0.982	0.956	0.912

According to the confusion matrix, out of 240 samples of the linear model, 24 samples were misidentified, and the highest error was for the 15^th^ day, when only 30 out of 45 data sets were correctly detected. According to the performance table for the C-SVM model, the values of Accuracy, Precision, and Recall were 0.97, 0.93, and 0.91, respectively.

### ANN results

**Fig 8:**
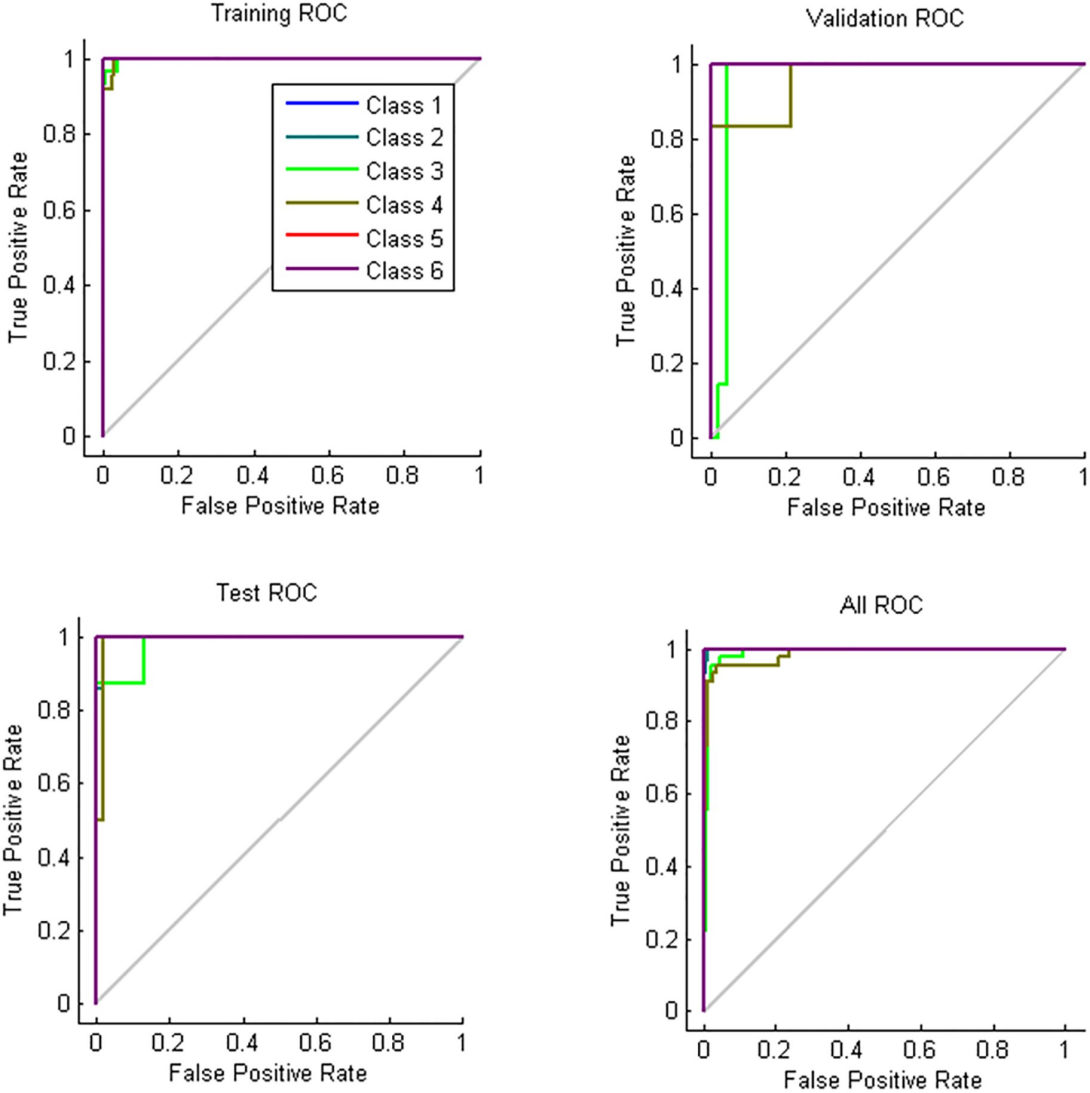
Receiver operating characteristic (ROC) curve for the ANN model developed. Abbreviations: Class1 = First day, Class2 = 15^th^ day, Class3 = 30^th^ day, Class4 = 45^th^ day, Class5 = 60^th^ day, and Class6 = 75^th^ day.

Table 8 presents the statistical data for the ANN model developed based on the e-nose signals as input (number of sensors) and storage periods as targets; therefore, the developed model contains 9 neurons in the input layer and 6 neurons in the output layer. Of the total data, 70%, 15%, and 15% were used for training, validation, and testing, respectively. The results are shown in Table 8. The developed models were evaluated in terms of correct classification rate (CCR) and root mean square error (RMSE). According to the results obtained for the three potato cultivars, the topology of 9-5-6 led to the best results (Fig 8). The accuracy of the artificial neural network method was very high, that is, its accuracy for identifying the shelf life of the Agria, Santé, and Sprite cultivars was 100%. Considering all samples, the topology of 9-5-6 led to values of 0.963 and 0.0723 for Train and 0.944 and 0.0166 for Test. The total detection accuracy was 96%. The confusion matrix and performance parameters of this network are presented in Tables 9 and 10. As the lower performance value of the training phase (when compared to the experimental phase) suggests, there was no sign of under/overfitting.

**Table 8:** Artificial neural network results.

Row	Topology	Train		Test		CCR (%)*
		RMSE	R^2^	RMSE	R^2^	
Agria	9-3-6	0.0500	0.796	0.0870	0.628	79
	9-4-6	0.0247	0.901	0.0357	0.862	90
	9-5-6	0.0000	0.999	0.0001	0.999	100
	9-6-6	0.0001	0.995	0.0014	0.994	99
	9-7-6	0.0035	0.987	0.0344	0.987	98
Santé	9-3-6	0.0051	0.794	0.0692	0.708	79
	9-4-6	0.0278	0.894	0.0354	0.851	89
	9-5-6	0.0000	0.999	0.0001	0.999	100
	9-6-6	0.0001	0.999	0.0011	0.999	99
	9-7-6	0.0007	0.997	0.0021	0.906	99
Sprit	9-3-6	0.0499	0.800	0.0617	0.746	73
	9-4-6	0.0259	0.901	0.0302	0.884	87
	9-5-6	0.0000	0.999	0.0001	0.999	100
	9-6-6	0.0045	0.983	0.0079	0.971	98
	9-7-6	0.0021	0.992	0.0043	0.984	99
All potato cultivars	9-3-6	0.0497	0.801	0.0705	0.701	80
	9-4-6	0.0274	0.895	0.397	0.844	89
	9-5-6	0.0723	0.963	0.0166	0.944	96
	9-6-6	0.0093	0.956	0.0126	0.933	95
	9-7-6	0.0094	0.947	0.0158	0.928	94

*The value of CCR was obtained from the confusion matrix.

**Table 9:** Confusion matrix for all group of potato based on storage time classification using the ANN statistical methods.

Topology	Storage time	First day	15^th^ day	30^th^ day	45^th^ day	60^th^ day	75^th^ day
9-5-6	First day	45	0	0	1	0	0
	15^th^ day	0	40	0	0	0	0
	30^th^ day	0	3	43	3	0	0
	45^th^ day	0	2	2	41	0	0
	60^th^ day	0	0	0	0	45	0
	75^th^ day	0	0	0	0	0	45

**Table 10:** Performance parameters of the SVM models for all potato cultivars.

Topology	Storage time	Accuracy	Precision	Recall	Specificity	AUC	F score
9-5-6	First day	0.996	0.978	1.000	0.996	0.987	0.989
	15^th^ day	0.981	1.000	0.889	1.000	1.000	0.941
	30^th^ day	0.970	0.878	0.956	0.973	0.925	0.915
	45^th^ day	0.970	0.911	0.911	0.982	0.947	0.911
	60^th^ day	1.000	1.000	1.000	1.000	1.000	1.000
	75^th^ day	1.000	1.000	1.000	1.000	1.000	1.000
Average per class		0.986	0.961	0.959	0.992	0.977	0.959

The receiver operating characteristic curve (ROC) in Fig 8 shows the actual positive rates (sensitivity) and the false-positive rates (specificity) in the classification of e-nose readings based on the 6 periods of potato storage. As it can be seen, the third and fourth periods of storage were less sensitive and accurate than others. The lowest precision (0.878) was in the third period, while the lowest accuracy for the third and fourth period was equal to 0.97. The lowest specificity (0.973) was observed in the third period.

**Fig 9:**
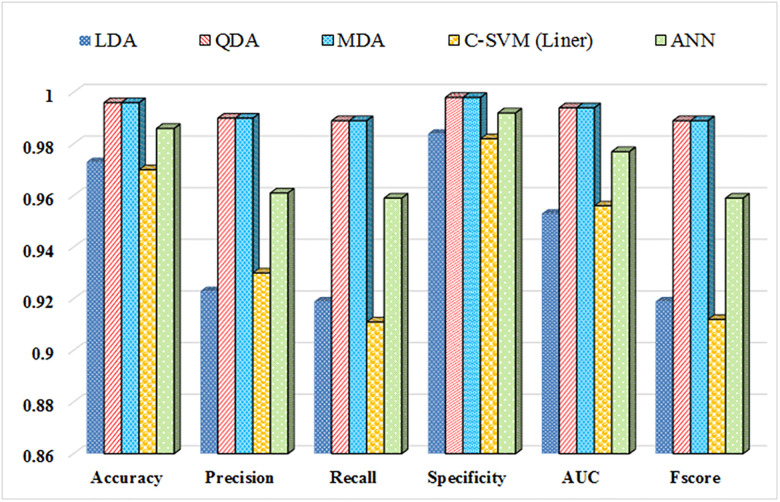
Average performance parameters of the statistical models in the classification of potato sample data. Data comparisons are derived from e‐nose VOC emission headspace volatile analyses, according to the storage time applied in the test plots.

Fig 9 summarizes the classification accuracy for the 6 potato storage periods. Among the tested models, the MDA and QDA models showed the best performance and highest accuracy in classifying the potato samples. The highest value of recall in QDA and MDA methods was 98.9% while the lowest value was in the SVM method (0.911). The three methods (QDA, MDA, and ANN) had the highest accuracy. Nonetheless, the LDA and SVM methods offered lesser accuracy. The mean performance parameters of the QDA and MDA models were exactly equal. In general, the overall accuracy of all models was high, and the combination of e-nose and chemometrics methods can be effectively used to classify the quality variations of potatoes in future programs.

## Discussion

As potatoes are harvested in a relatively short time, but consumed for a long time, their high post-harvest losses have become a major limiting factor in extending their application. Therefore, proper storage of potatoes to reduce the post-harvest losses is of crucial importance. Today, storage in a refrigerator is one of the most common and effective ways to maintain the post-harvest quality of sweet potato tubers while keeping their freshness and sweetness. Various factors during the storage, such as light, temperature, and humidity can reduce the quality of potatoes. Accurate detection of declined quality of potatoes during the storage period can prevent its commerciality problems. Therefore, selecting the potatoes with declined quality is important to guide the distributors and retailers toward better decision-making strategies. In this regard, the detection of VOC in potatoes will offer promising practical prospects for controlling the refrigerator conditions during the storage period. However, a limited number of studies have applied the e-nose technology to predict variations of potato quality.

Several methods have been proposed for estimating and predicting the storage and storage period of agricultural and food products. In a study, gas chromatography-mass spectrometry-based metabolomics was used to identify the applicable biomarkers to predict potato storage and germination. Sucrose, phosphate, and amino acids were used as valid contributing biomarkers to predict in a validation field experiment. With the PLS regression model, they achieved 78–96% accuracy for predicting potato storage and germination [58]. whose accuracy was similar to the results of our research with the PCA, DA and SVM methods. In another study, the TSS (total soluble solids) and DMC (dry matter content) of cucumber were used as indicators of its shelf life. This study showed a gradual decrement in the TSS and DMC of cucumbers during storage. The longer the time from harvest to consumption, the greater the decrease. Further, the relationship between TSS and DMC was linear from collection to aging. Storage time, as well as the TSS and DMC contents at harvest time affect the marketability of cucumber during harvest and under refrigerated conditions. Higher TSS and DMC contents at harvest indicate longer shelf life [59]. In another study, the shelf life of potatoes was modeled in evaporative cooling storage, using an improved bin for potato storage. The results showed a lower percentage of cumulative spoilage in this method, compared to the traditional methods of farmers. Potato shrinkage was almost the same in both methods. The sugar content of the modified storage method was lower during the storage period and showed a smaller increase compared to conventional approaches [60]. In another study, sprout suppressants were employed to increase the shelf life of potato tubers, stating the key role of proper germination management in the prevention of tuber germination. Moreover, to increase the storage and transportation of potatoes, maintenance of dormancy or the use of sprout suppressors are required [61]. The researchers used a combination of sonication with anti-browning treatments as a strategy to increase the shelf life of freshly sliced potatoes. In this study, two anti-browning solutions were assessed to prevent browning and increase the shelf life of freshly sliced potatoes. At a temperature of 4°C, NatureSeal^®^ successfully prevented the spoilage of sliced potatoes [62]. An investigation to reveal the potential relationship between the bacterial community and quality attributes of vacuum-packaged peeled potatoes, the bacterial community dynamics, visual quality, organic acids, flavor and volatile organic compounds (VOCs) during 12 days of storage under 10 °C were studied with the help of an electronic nose. The researchers stated that the results of this study have great practical significance for prolonging the shelf life of fresh-cut agricultural produce [63]. In another research, maturity indicators (including SSC for physiological maturity and sucrose, glucose and fructose content for chemical maturity) were investigated as quality predictors in Asterix and Saturna potato tubers during and after storage. Potato quality parameters measured during storage included dry matter content, sucrose, glucose and fructose content, weight loss and frying color. Linear regression models were able to explain a high proportion of variation (R^2^ ≥ 0.88) [64]. However, their accuracy was far lower than the accuracy of this study results. Zhao et al. [65] presented a model to predict the shelf life of fresh-cut potatoes at different storage temperatures. They placed fresh-cut potatoes in chlorine dioxide solution (100 mg/L), citric acid solution (1.5%) respectively) and potassium sorbate solution (0.1%) and then wrapped them with polyvinylidene chloride plastic film. Then they were kept at 4 temperatures of zero, 4, 7 and 10 degrees Celsius. They studied the quality-variation relationship between samples over time and temperature, mainly with weight loss, PPO (*Polyphenol oxidase*) activity and the total number of colonies. The results show that weight loss, PPO activity and an unlimited number of colonies increase with increasing time and temperature. By comparing the predicted values ​​with the experimental values, they stated that the shelf life prediction model of PPO activity could provide a more accurate prediction of the shelf life of fresh-cut potatoes at different temperatures (R^2^ = 0.882÷0.988). The accuracy of the results of this research was very similar to the results of our research.

## Conclusions

The application of an e-nose in combination with machine learning modeling could be a cost-effective and rapid approach to monitor the qualitative changes of potatoes during the storage period. The VOCs emitted from potatoes during the storage period can vary, depending on their quality. This provides a non-destructive method to monitor the quality of the potatoes. E-noses encompass a hardware section and detection and pattern algorithms. Compared to conventional methods, such as GC-MS, e-noses are non-destructive as well as provide a fast and cost-effective option for several applications. However, the use of e-noses for qualitative monitoring is at an early stage of development. Thus, advanced, real-time, cost-effective, and portable approaches are highly welcome for detection in various storage stages. A portable e-nose system equipped with sensitive gas sensors and pattern detection could be a novel approach to meet these requirements. It can also offer several advantages over the conventional methods and resolve their field challenges. Further studies are required to verify the validity and repeatability of e-nose systems developed in diverse crops and open fields.
